# Integrating an Enhanced Sampling Method and Small-Angle X-Ray Scattering to Study Intrinsically Disordered Proteins

**DOI:** 10.3389/fmolb.2021.621128

**Published:** 2021-04-15

**Authors:** Chengtao Ding, Sheng Wang, Zhiyong Zhang

**Affiliations:** ^1^ MOE Key Laboratory for Membraneless Organelles and Cellular Dynamics, National Science Center for Physical Sciences at Microscale, Division of Life Sciences and Medicine, University of Science and Technology of China, Hefei, China; ^2^ Tencent AI Lab, Shenzhen, China

**Keywords:** IDPs, biological function, MD simulation, sampling, integrative modeling

## Abstract

Intrinsically disordered proteins (IDPs) have been paid more and more attention over the past decades because they are involved in a multitude of crucial biological functions. Despite their functional importance, IDPs are generally difficult to investigate because they are very flexible and lack stable structures. Computer simulation may serve as a useful tool in studying IDPs. With the development of computer software and hardware, computational methods, such as molecular dynamics (MD) simulations, are popularly used. However, there is a sampling problem in MD simulations. In this work, this issue is investigated using an IDP called unique long region 11 (UL11), which is the conserved outer tegument component from herpes simplex virus 1. After choosing a proper force field and water model that is suitable for simulating IDPs, integrative modeling by combining an enhanced sampling method and experimental data like small-angle X-ray scattering (SAXS) is utilized to efficiently sample the conformations of UL11. The simulation results are in good agreement with experimental data. This work may provide a general protocol to study structural ensembles of IDPs.

## Introduction

It has been recognized that a large segment of the human proteome comprises intrinsically disordered proteins (IDPs) that lack stable secondary and tertiary structures under physiological conditions (Colak et al., 2013; Kulkarni and Uversky, 2019). IDPs play important roles in a multitude of crucial biological functions despite their lack of a stable structure, such as cell cycle regulation, molecular recognition, and signal transduction (Dunker et al., 2005; Uversky et al., 2005). According to previous work, IDPs are involved in the majority of human cancer (Iakoucheva et al., 2002) and many chronic diseases like cardiovascular disease (Cheng et al., 2006), neurodegenerative diseases (Uversky, 2009; Uversky, 2014), and type 2 diabetes (Du and Uversky, 2017).

Although researchers continue to discover the functional importance of IDPs, it remains difficult to explore the structure-function relationship because getting the high-resolution structures of IDPs remains elusive. Since an IDP is generally not stable in one conformational state, these classical technologies of structural biology, including X-ray crystallography and cryo-EM, cannot determine its atomic-resolution structure. Alternatively, structural information on the ensemble average of the IDP is available by techniques like nuclear magnetic resonance (NMR) (Dunker and Oldfield, 2015), small-angle X-ray scattering (SAXS) (Bernado and Svergun, 2012), and Förster resonance energy transfer (FRET) (LeBlanc et al., 2018).

In order to obtain structural details of IDPs, atomistic molecular dynamics simulation is a useful and complementary method for illuminating the molecular nature of IDPs’ conformational ensembles because it can provide spatial and temporal resolution unavailable from experiments (Potoyan and Papoian, 2011; Burger et al., 2014; Granata et al., 2015; Bhowmick et al., 2016). Despite the significant progress made, a sampling problem remains in MD simulations of IDPs. The conformational space of an IDP is generally very large, so conventional MD simulations at a timescale of microseconds (μs) cannot capture all the states adequately. To tackle this problem, many enhanced sampling methods have been developed, which achieve good sampling by modifying potential energy function (Hamelberg et al., 2004) or increasing the temperature of barrier regions (Zhang et al., 2003; Hu et al., 2012). In recent years, a new kind of sampling techniques has been proposed, which are built on iterative multiple independent MD (MIMD) simulations (Harada and Kitao, 2013, Harada and Kitao, 2015; Shkurti et al., 2019; Yuan et al., 2020; Zhang and Gong, 2020). Such a method generally contains many cycles, and each cycle consists of a number of short MIMD simulations starting from selected seed conformations. The sampling efficiency would depend on the strategy of selecting seeds, and different criteria have been tried (Harada and Shigeta, 2018).

Many studies have shown the possibility of combining experimental data and computational simulations to interpret structural dynamics of large biomolecules in a solution that is called integrative modeling (Braitbard et al., 2019). There are various integrative modeling techniques for the interpretation of different structural data (Bonomi et al., 2017; Saltzberg et al., 2019; Orioli et al., 2020), which can be divided into two categories: refining-while-sampling and the screening-after-sampling (Zhang et al., 2015). A refining-while-sampling method directly adds an extra pseudo energy term based on the experimental data and then a conformation or an ensemble is simulated by optimizing the energy (Zheng and Tekpinar, 2011; Bjorling et al., 2015). In a screening-after-sampling method, a structure pool of the biomolecule is firstly sampled without experimental restraints, and then a reweighting method acts on these conformations to optimize their weights in order to fit the experimental data well (Bottaro et al., 2020). An ensemble containing a small number of conformations selected from the pool could be determined (Bernado et al., 2007; Curtis et al., 2012).

In this work, we propose a general strategy to study the conformations of IDPs. After choosing a suitable force field and water model for simulating IDPs, an integrative modeling procedure combining an enhanced sampling method based on iterative MIMD and SAXS data is used to sample conformations of IDPs efficiently. We present a case study on an IDP called unique long region 11 (UL11), an RNA-binding protein that is one of the conserved outer tegument components from herpes simplex virus 1 (HSV-1) (Bowzard et al., 2000; Metrick et al., 2020).

HSV-1 contains a unique tegument layer sandwiched between the capsid and lipid envelope, including 24 tegument proteins (McLauchlan and Rixon, 1992). UL11 is the smallest tegument protein with only 96 amino-acid residues (MacLean et al., 1989; Bowzard et al., 2000). UL11 and its homologs have been found to play crucial roles in efficient viral replication (MacLean et al., 1992; Baird et al., 2010) and tegument assembly (Owen et al., 2015). However, the mechanistic understanding of its role in these processes is limited due to the lack of knowledge of its biochemical and structural properties. A recent article (Metrick et al., 2020) has suggested that UL11 is an IDP in solution, which can undergo liquid–liquid phase separation (LLPS) *in vitro*. Analysis of experimental SAXS data showed that the protein is highly dynamic. Here, we aim to construct an atomic structural ensemble of UL11 that is in agreement with the available experimental data.

## Materials and Methods

### An Initial Atomic Model of UL11

The UL11 construct used in this work is called UL11-Stll (Metrick et al., 2020), which is the UL11 sequence (96 residues) plus a small C-terminal Strep-tag II (Stll) including eight residues (WSHPQFEK). We used this 104-residue construct, on which the SAXS experiment was conducted. In the following, we call this construct UL11 for simplicity.

**FIGURE 1:**
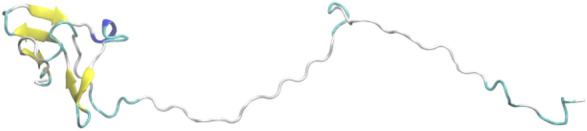
An atomic model of UL11 predicted by tFOLD.

According to a prediction from the FoldUnfold server (http://bioinfo.protres.ru/ogu), many residues of UL11 are predicted to be disordered, except for some N-terminal residues that are natively folded (Metrick et al., 2020). We predicted an atomic model of UL11 using the tFOLD server (https://drug.ai.tencent.com/console/cn/tfold) (Figure 1). There are some β-strands at the N-terminus (residues 11–14, 17–20, 24–27, 39–41, and 44–47), while the other regions are disordered till the C-terminal end. The tFOLD model is consistent to the prediction of the disorder, so we used it as a starting structure for simulations.

### Simulation Details

In this work, all-atom conventional MD (cMD) simulations and accelerated MD (aMD) simulations were conducted using the Amber20 package.

#### Conventional MD (cMD) Simulation

It has been recognized that, in MD simulations using those traditional force fields and water models, IDPs may become over-compact. Therefore, combinations of new force fields and water models have been proposed to address this issue (Kuzmanic et al., 2019). In this work, we used the A99SB force field in combination with a 4-point OPC water model (Izadi et al., 2014). It has been reported that this A99SB/OPC combination is suitable for simulating conformations of IDPs (Shabane et al., 2019).

The system was built via the LEaP module (Case et al., 2005). The OPC waters (Izadi et al., 2014) were added to a truncated octahedral box with a minimal distance of 10.0 Å between the solute and the box boundary. 102 Na^+^ and 98 Cl^−^ ions were added by replacing water molecules to balance the charge on the system and bring the salt concentration to about 100 mM NaCl. The box size is 1.66 × 10^6^ Å^3^, with 205,909 atoms in total. To remove bad contacts, the waters and ions were initially minimized for 2,000 steps using the steepest descent method for the first 1,500 steps and then the conjugate gradient for the last 500 steps, with the position of protein fixed (force constant was 500 kcal mol^−1^ Å^−2^). In the second energy minimization, the restraints on the protein were removed. This stage was conducted for 2,500 steps, using the steepest descent method in the first 1,000 steps and then the conjugate gradient algorithm for the last 1,500 steps. After that, a heat-up MD was run at a constant volume. The system was heated from 0 to 300 K for 100 ps with a weak restraint of 10 kcal mol^−1^ Å^−2^ on the solute. A free MD simulation of 150 ns was carried out under the NPT condition utilizing the GPU-accelerated pmemd.cuda code. The temperature was regulated using the Langevin dynamics with a collision frequency of 1.0 ps^−1^ (Pastor et al., 1988). Pressure was controlled with isotropic position scaling at 1 bar with a relaxation time of 2.0 ps. All the bonds involving hydrogen atoms were constrained using the SHAKE algorithm (Ryckaert et al., 1977). A 2 fs integration step was used. Van der Waals interactions outside the cutoff distance were approximated via a continuum model (vdwmeth = 1) (Izadi et al., 2014; Izadi and Onufriev, 2016). The long-range electrostatic interaction was calculated using the PME method (Muller et al., 1996) with a 10 Å cutoff for the range-limited nonbonded interaction.

#### Accelerated MD (aMD) Simulation

The aMD (Muller et al., 1996) introduces a boost potential, ΔV(r), to the original potential energy V(r) when the latter is below a threshold energy E:

$$\Delta V\left(r\right)=\{\begin{array}{cc} 0, & \text{V}\left(r\right)\geq E,\\ {\left[\frac{\left(E-\text{V}\left(r\right)\right)}{\alpha +\left(E-\text{V}\left(r\right)\right)}\right]}^{2}, & \text{V}\left(r\right)<E. \end{array}$$ (1)

where α is a factor that tunes the depth of the modified energy basins. Boosting potentials were applied to both the total potential and the individual dihedral energy term. The aforementioned 150 ns cMD simulation was used to estimate the aMD parameters. In the cMD trajectory, the average total potential energy was −641,138 kcal mol^−1^ and the average dihedral energy was 1,068 kcal mol^−1^. UL11 has 104 residues and the simulated system consists of 205,909 atoms. The following parameters were set based on the above information:

E (tot) = −641,138 kcal mol^−1^ + (0.2 kcal mol^−1^ atom^−1^ × 205,909 atoms)≈−599,956 kcal mol^−1^
α (tot) = 205,909 atoms × 0.2 kcal mol^−1^ atom^−1^≈41,182 kcal mol^−1^
E (dih) = 1,068 kcal mol^−1^ + (3.5 kcal mol^−1^ residue^−1^ × 104 residues)≈ 1,432 kcal mol^−1^
α (dih) = 0.2 × (3.5 kcal mol^−1^ residue^−1^ × 104 residues)≈73 kcal mol^−1^

With these parameters, a 150 ns aMD simulation was conducted. All the other parameters were the same to the aforementioned cMD simulation.

### The Strategy of Integrative Modeling

**FIGURE 2:**
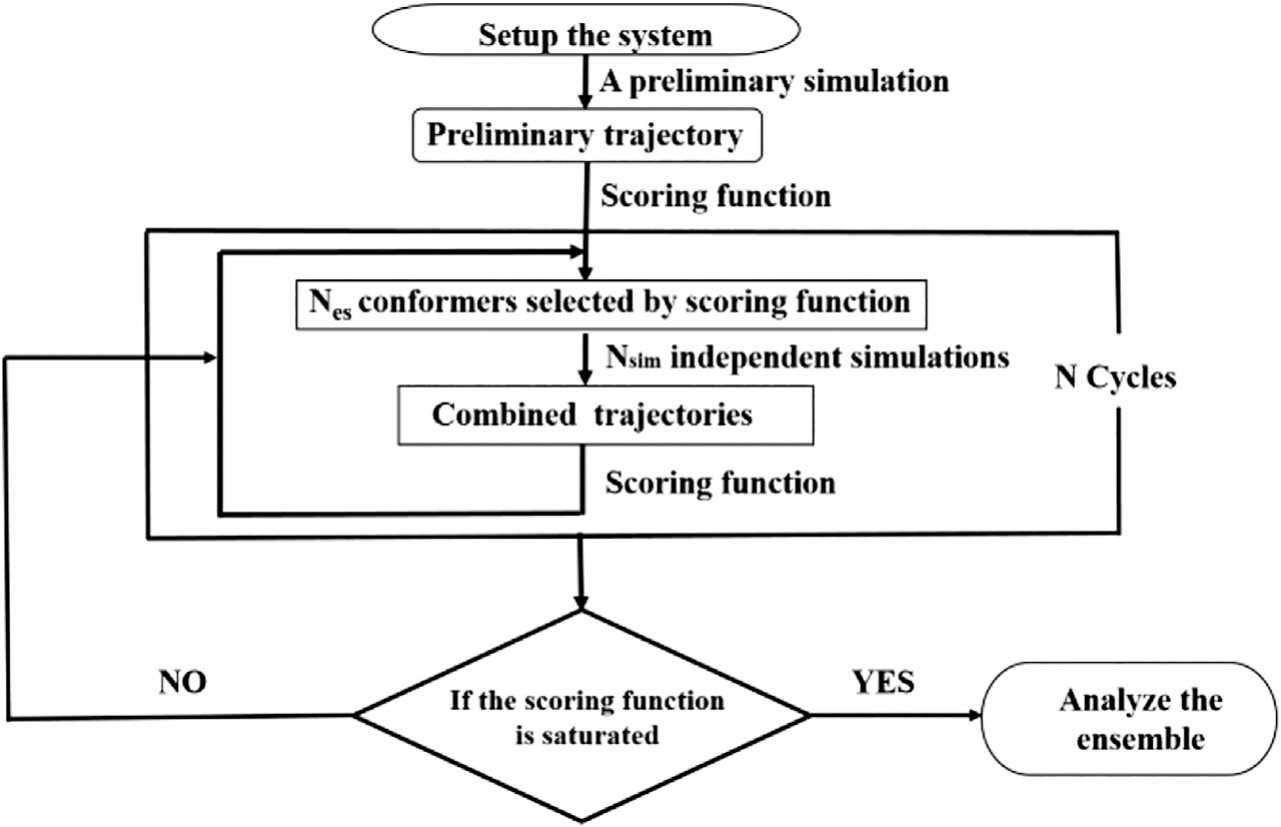
Flowchart of integrative modeling that is a modification from Figure 1 in (Cheng et al., 2017).

We have previously developed a method called SAXS-oriented ensemble refinement (SAXS-ER) (Cheng et al., 2017), and the flowchart is as follows (Figure 2). The code is available at https://github.com/pcheng27/SAXS-ER/tree/v1.1.

1) Set up the system starting from an initial structure of the biomolecule, and perform a preliminary simulation. Any simulation method can be utilized, such as atomistic MD simulations, enhanced sampling techniques, or coarse-grained modeling. In this work, we are studying an IDP, and the sampling is challenging. Therefore, aMD simulations are carried out using the most updated code of pmemd.cuda in the Amber20 package.
2) Calculate the scoring function and obtain an ensemble of conformers with the best score. The number of conformers in the ensemble is N_es_. In this work, the scoring function is χ^2^ between the calculated SAXS profile of the ensemble and the experimental SAXS profile. More details will be introduced in the “Ensemble Optimization Method” section.
3) Starting from the N_es_ conformers selected by scoring function, N_sim_ (=N_es_)-independent simulations are carried out. Multiple independent short-time simulations may achieve a better sampling than a single long-time simulation. All the trajectories are combined.
4) Repeat steps 2 and 3 for N cycles. Analyze all those cycles with the saturated scoring function.

### SAXS Data

**FIGURE 3:**
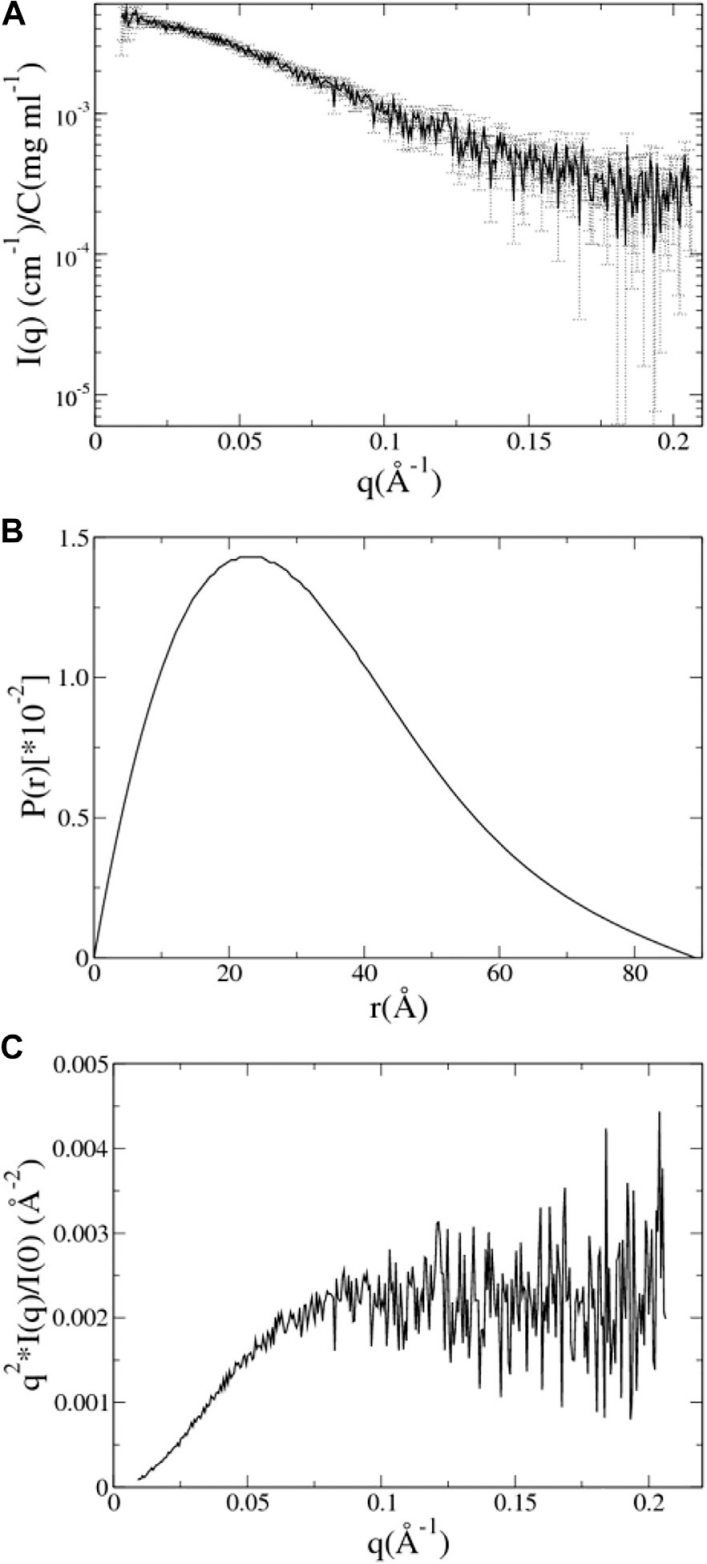
SAXS data analysis of UL11. **(A)** The experimental SAXS profile of UL11 is shown with errors. **(B)** The pair distance distribution function (PDDF) is normalized so that the sum under the curve is 1. **(C)** Kratky plot.

The SAXS data of UL11 were taken from SASBDB (www.sasbdb.org) with the ID SASDEX4. All the experimental details and analyzed results can be found in the database and the published article (Metrick et al., 2020). In this work, we took the data points with q from 0.009 to 0.206 Å^-1^ (q=4π⁡sin⁡θ/λ, where 2θ is the scattering angle and λ is the wavelength of 1.246 Å), and the signal–noise ratios in this range are essentially larger than 2.0 (Figure 3A). The radius of gyration (R_g_) of the protein was estimated to be 24.1 ± 1.7 Å by Guinier analysis using the autoRg program in the ATSAS package (Franke et al., 2017). The pair distance distribution function (PDDF) was calculated by GNOM (Semenyuk and Svergun, 1991) using the maximum dimension (D_max_) of 89.0 Å as input. The normalized PDDF is asymmetrical and tailed off to a large distance (Figure 3B), which resembles the shape of an elongated ellipsoid (Mertens and Svergun, 2010). Therefore, the protein should be able to take extended conformations in the solution that can be disordered. The Kratky plot (Figure 3C) also supports that the protein is an IDP with partially folded regions.

CRYSOL (Svergun et al., 1995) was used to compute the theoretical SAXS profile of a known atomic structure in PDB format, and then autoRg was run on the SAXS profile to estimate the R_g_ of the structure. The CaPP software, available at github.com/Niels-Bohr-Institute-XNS-StructBiophys/CaPP, was used to calculate PDDF from these PDB files.

### Ensemble Optimization Method

A structural ensemble was obtained by the ensemble optimization method (EOM) (Bernado et al., 2007). EOM was used to select a small number of representative conformations from a pool containing lots of conformations of UL11 in order to fit the experimental SAXS data. The scoring function of EOM is as follows:

$$\chi^{2}=\frac{1}{K-1}\sum_{i=1}^{K}{\left[\frac{\mu I\left(q_{i}\right)-I_{\exp}\left(q_{i}\right)}{\sigma \left(q_{i}\right)}\right]}^{2},$$ (2)

where K is the number of data points in the SAXS profile and σ(q) are experimental errors. For every conformation in the ensemble, its theoretical scattering profile is computed. I(q) is the average of them, and μ is a scaling factor.

A new version of EOM called EOM2 (Tria et al., 2015) was used to compute the scoring function (Eq. 2) and pick the ensembles. In the original SAXS-ER using EOM2 (Cheng et al., 2017), the program automatically determined the ensemble size in each cycle that was generally small. An IDP should be represented by an ensemble containing more conformers than folded proteins. Therefore, in this work, we used an option of fixing the ensemble size to a relatively large number like 24 when running EOM2 in each cycle.

## Results and Discussion

**FIGURE 4:**
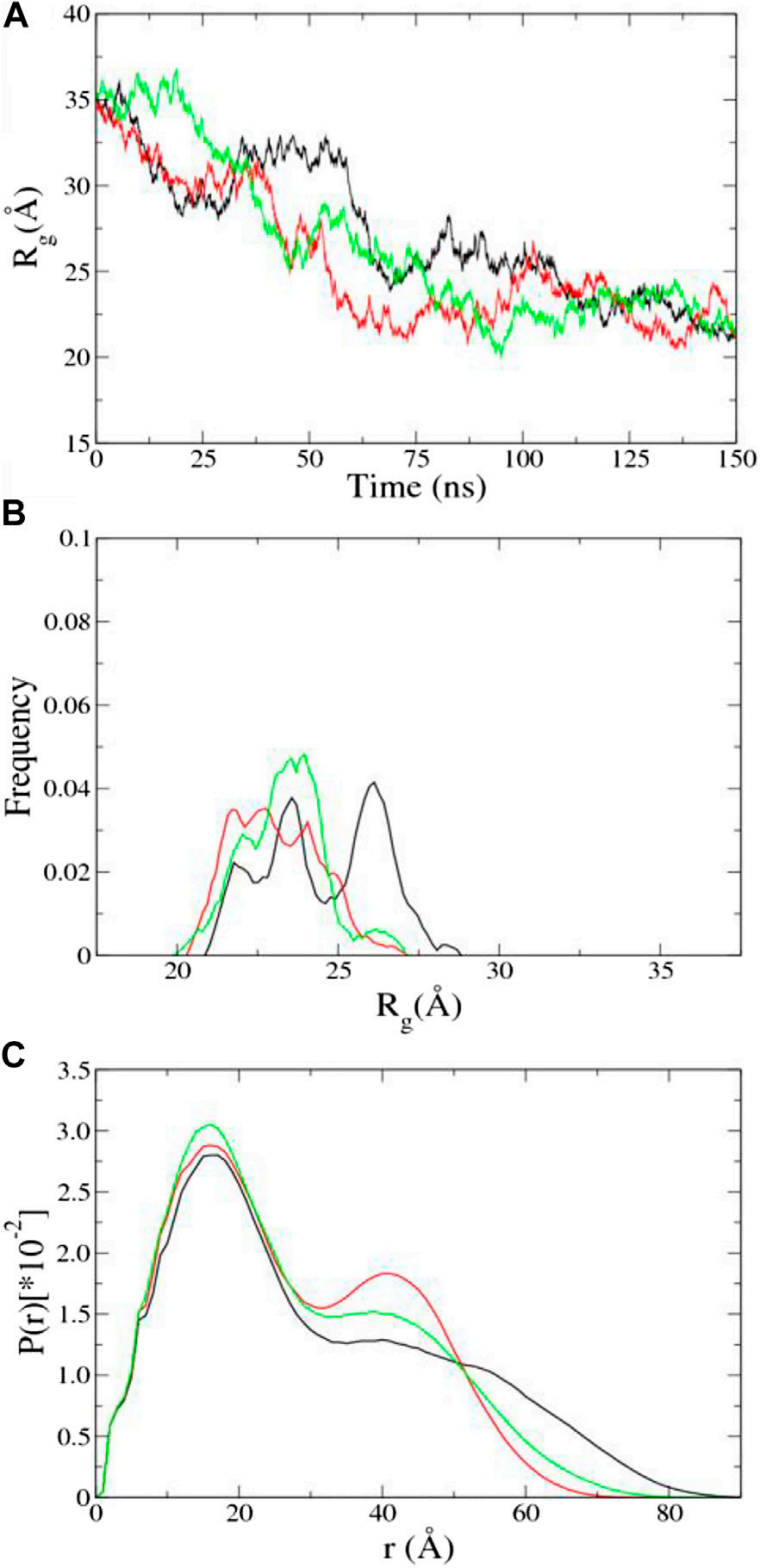
Results of aMD using A99SB/OPC. **(A)** Time evolution of R_g_. **(B)** R_g_ distribution in the last 80 ns aMD simulations. **(C)** Ensemble-averaged PDDF in the last 80 ns aMD simulations. The three independent simulations are shown in different colors.

***aMD of UL11 without Integrating the SAXS Data.*** Three independent aMD simulations, each of 150 ns, were conducted. We converted a trajectory into sequentially individual PDB files; then CRYSOL and autoRg were run to obtain R_g_ of each atomic structure as described in the “SAXS Data” section. The initial structure of UL11 (Figure 1) is extended with R_g_ of 35.2 Å. In the first 70 ns of the aMD simulations, the protein is equilibrating with a clear tendency of R_g_ decrease (Figure 4A), and then the R_g_ values essentially fluctuate between 21.0 and 27.5 Å in the remaining simulations. According to the R_g_ distribution of the conformations in the last 80 ns (Figure 4B), they seem to show agreement with the experimental R_g_ of 24.1 ± 1.7 Å. We calculated the PDDF of each conformation in the last 80 ns of one trajectory and then plotted the ensemble-averaged PDDF (Figure 4C). The shape of the three ensemble-averaged PDDF curves is obviously not similar to that of the experimental PDDF (Figure 3B). That is to say, the aMD simulations at the time scale of 150 ns cannot adequately sample solution conformations of the IDP, which is the cause for the discrepancy between the simulated and the experimental PDDF. A straightforward way is to simply run longer simulations so that the protein could expand again and sample diverse conformations. However, it is not sure how long would be long enough to give a representative picture of the IDP. Therefore, we performed integrative modeling of UL11.

***Integrative Modeling of UL11.*** Starting from the same structural model (Figure 1), we conducted integrative modeling of UL11 using the protocol introduced in Figure 2. A cycle consisted of N_sim_ = 24 independent 200 ps aMD simulations using A99SB/OPC. In each aMD simulation, a conformation was recorded every 1 ps, so a structural pool containing 4,800 conformations was generated in one cycle. By fitting the experimental SAXS data of UL11, EOM2 selected an ensemble with the size of N_es_ = 24 from the pool. Starting from these conformations, the next cycle of multiple independent simulations was run. We carried out 30 cycles, so the total simulation time was 144 ns (200 ps × 24 aMD × 30 cycles).

**FIGURE 5:**
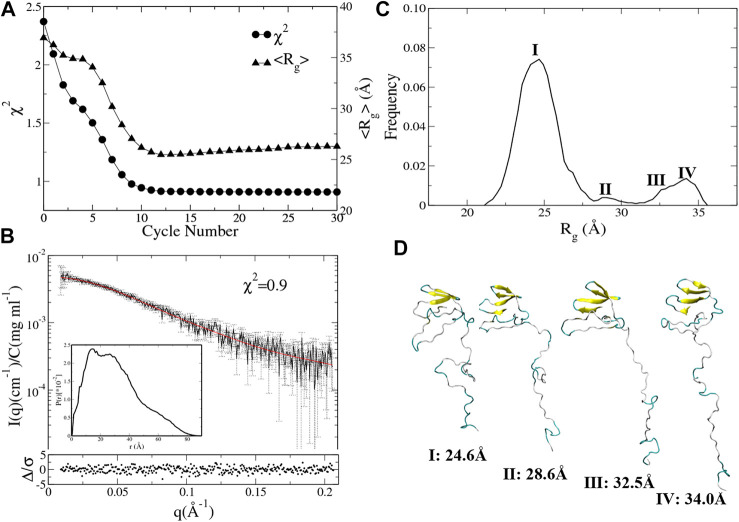
Integrative modeling from an extended structure of UL11 (Figure 1). **(A)** The minimal χ^2^ (circle) and the corresponding <R_g_> (up-triangle) at each cycle. **(B)** The back-calculated SAXS profile of the selected ensemble (red line) is fitted to the experimental data (black line with errors). The lower plot shows the error-weighted residual of the model fitting. The inset is the normalized ensemble-averaged PDDF. **(C)** The distribution of R_g_ values calculated from the ensembles after the 11th cycle. **(D)** Representative structures according to the R_g_ distribution.

The χ^2^ and the average R_g_ (<R_g_>) of the ensemble are plotted against the cycle number (Figure 5A). The initial model of UL11 is very extended (Figure 1); the EOM ensemble generated at cycle 0 cannot fit the experimental SAXS data well, with a χ^2^ of 2.3. It is found that χ^2^ decreases relatively fast in the first eight cycles (from 2.3 to 1.0), and then it slowly converges to about 0.9 after the 10th cycle (Figure 5A, circle). When looking at the time evolution of the <R_g_> (Figure 5A, up-triangle), it converges to 25.5 Å after 12 cycles, that is in good agreement with the estimated R_g_ (24.1 ± 1.7 Å) from the experimental SAXS data (Figure 3A). Therefore, we plotted the calculated SAXS profile of the ensemble at the 12th cycle and its error-weighted residual (Figure 5B). The residuals are defined as $\left(I_{exp}\left(q\right)-I_{calc}\left(q\right)\right)/\sigma_{exp}\left(q\right)$, corresponding to the difference between the experimental and the computed intensities weighted by the experimental uncertainty (Carter et al., 2015; Trewhella et al., 2017). The residual difference plot is flat, which indicates that the results are in good agreement with the data. The inset is the normalized average PDDF of the ensemble, which has a similar shape to the experimental PDDF (Figure 3B).

To characterize conformations consistent with the SAXS data, we analyzed the R_g_ distribution of all the ensembles after the 11th cycle (Figure 5C). There is a major peak with the R_g_ value around 24.6 Å, a minor peak located between 27.5 and 30.0 Å, and two more peaks with the R_g_ values larger than 30.0 Å that do not appear in the 150 ns aMD simulations (Figure 4B). A representative structure of each peak is shown in Figure 5D. One can clearly see several states of UL11, which correspond to relatively compact, intermediate, and extended conformations, respectively.

**FIGURE 6:**
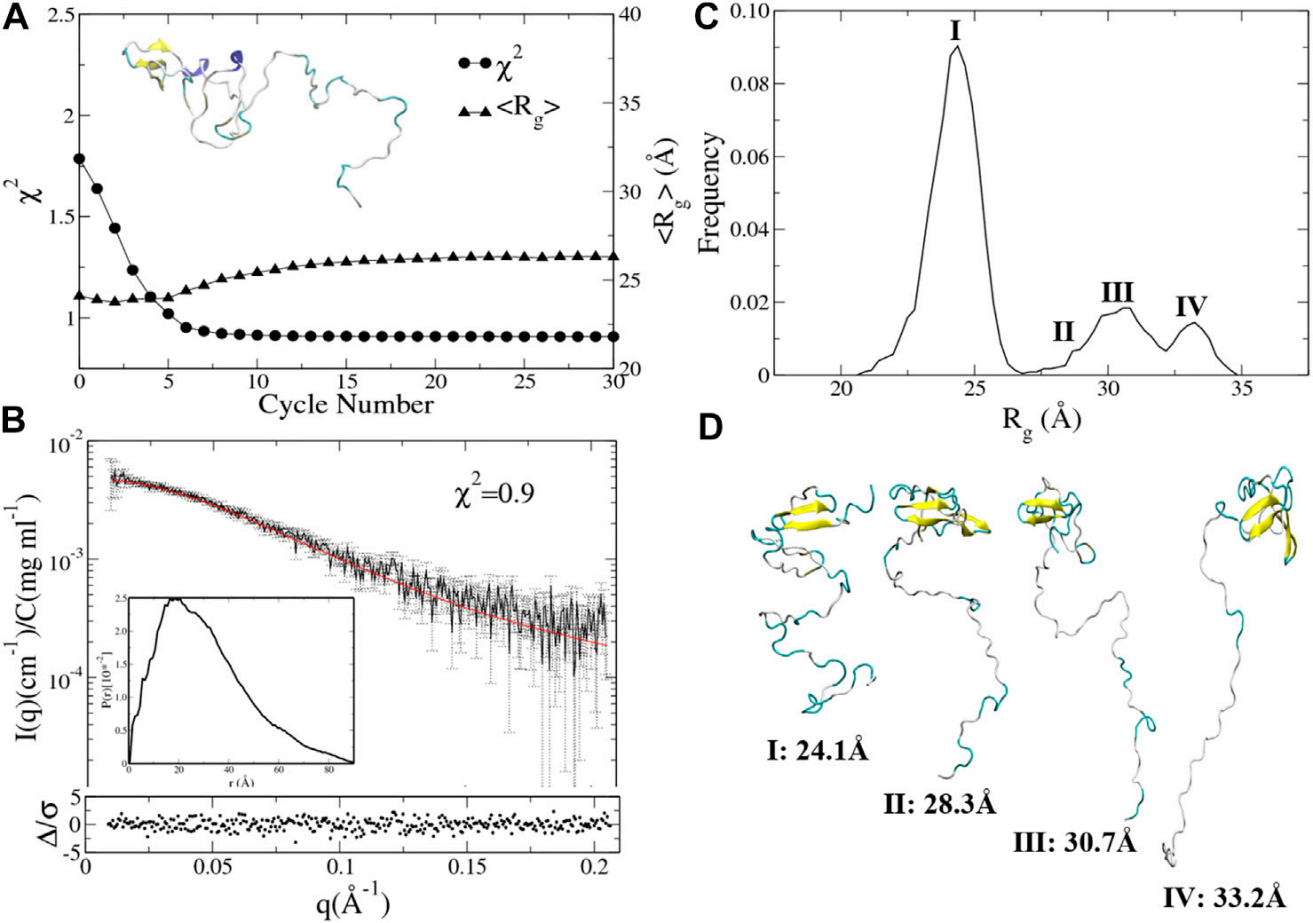
Integrative modeling from a relatively compact structure of UL11. **(A)** The minimal χ^2^ (circle) and the corresponding <R_g_> (up-triangle) at each cycle. The inset is the starting structure. **(B)** The back-calculated SAXS profile of the selected ensemble (red line) is fitted to the experimental data (black line with errors). The lower plot shows the error-weighted residual of the model fitting. The inset is the ensemble-averaged PDDF. **(C)** Distribution of R_g_ values calculated from the ensembles after the 11th cycle. **(D)** Representative structures according to the R_g_ distribution.

To test the reproducibility of the results, we also conducted the integrative modeling starting from a relatively compact structure of UL11 (inset in Figure 6A) taken from the 150 ns cMD simulation using A99SB/OPC. χ^2^ and <R_g_> of the ensemble are plotted against the cycle number (Figure 6A). χ^2^ of the ensemble at cycle 0 is 1.8, and only after seven cycles, it converges to 0.9 (Figure 6A, circle). < R_g_> of the ensemble at cycle 0 is 23.6 Å, and it converges to 25.8 Å after 11th cycles (Figure 6A, up-triangle). We plotted the calculated SAXS profile of the ensemble at the 12th cycle and its error-weighted residual (Figure 6B). The residual difference plot between the experimental and the computed I(q) is flat, which indicates that the results fit with the data. The normalized ensemble-averaged PDDF is in agreement with the experimental curve (Figure 3B). The R_g_ distribution of all the ensembles after the 12th cycle also indicates a major peak around 24.1 Å, a minor one between 27.5 and 30.0 Å, and two more peaks with the R_g_ values larger than 30.0 Å (Figure 6C). The representative structures of the peaks (Figure 6D) correspond to states of UL11 from the relatively compact, the intermediate, and to the extended conformations. It has been found that the two independent integrative models of UL11 starting from the different structures show fairly consistent results.

It is worth noting that the total time scale of the integrative modeling is only 144 ns, but it can achieve a more efficient sampling and better convergence than the 150 ns aMD simulations (Figure 4).

In a previous work (Metrick et al., 2020), the authors ran RANCH, an internal program of EOM2, to generate a coarse-grained structural pool using a simple exclusion energy term. Then EOM was applied to the pool to pick an ensemble by fitting the SAXS data. The ensemble also included states from compact to extended. Our results of integrative modeling support their study. However, our ensembles consist of atomic models generated by fine Amber force field and explicit water model, which should be physically more reasonable than those generated by RANCH. However, more experimental data would be needed to further validate these models.

## Conclusion

This work integrates an enhanced sampling method and experimental SAXS data to study IDPs. In our strategy, we first need to choose a combination of the force field and water model, such as A99SB/OPC, that is suitable for simulating IDPs, and then an enhanced sampling technique like aMD is taken. After that, integrative modeling is conducted based on iterative multiple independent simulations. Experimental data like SAXS are used to design a scoring function for screening conformations and thus guide the simulations toward an ensemble that fits the experimental data well. Therefore, we think this strategy of integrative modeling is well suited for investigating conformational ensembles of IDPs.

We have carried out the integrative modeling of UL11, which is important for efficient viral replication and tegument assembly. To the best of our knowledge, the understanding of its biochemical structure and mechanism is still limited, except for some coarse-grained structural information (Metrick et al., 2020). In this work, we have predicted an ensemble of atomic structures, which includes both the relatively compact and extended conformations of UL11. This ensemble is in agreement with the available experimental data and may provide information on the functional mechanism of UL11. It has been said that UL11 undergoes LLPS *in vitro* (Metrick et al., 2020). Our study on the monomer and the integrative modeling strategy may be helpful for future research on LLPS.

There are various tools for integrative modeling (Bonomi et al., 2017; Orioli et al., 2020), which use either the refining-while-sampling or the screening-after-sampling strategy. A refining-while-sampling method is efficient, but one needs to modify complicated simulation code to add an energy term for experimental restraints. In a screening-after-sampling method, although there is no need to change the simulation code, the postprocessing reweighting procedure would rely on adequately sampling conformations of the biomolecule, which is, however, a nontrivial issue for IDPs. Our method can be regarded as an iterative screening-after-sampling strategy, so we do not change the MD code. However, the sampling is still efficient because it is guided by the experimental data.

Our integrative modeling method has some other characteristics. The first is that the iterative multiple independent simulations are very suitable for parallel computing. In this work, 24 independent simulations are run simultaneously, but one can use more CPU/GPU if they are available. The second is the high adaptability. Any sampling methods and ensemble optimization methods can be easily implemented with minor modifications to the scripts. Last but not least, many experimental data may be integrated simultaneously as long as a proper scoring function is designed. One of the future improvements is to input multiple initial models at the beginning of the integrative modeling in order to sample the conformations of IDPs as adequately as possible.

## Data Availability

The raw data supporting the conclusions of this article will be made available by the authors, without undue reservation.
